# Solitary Retroperitoneal Neurofibroma Associated With Deep Vein Thrombosis in a 40-Year-Old Male

**DOI:** 10.7759/cureus.23587

**Published:** 2022-03-28

**Authors:** Osama Shahid, Raoon Khan, Mubasshar Shahid, Muhammad Taimour Khan, Maryam Iqbal

**Affiliations:** 1 Internal Medicine, Combined Military Hospital (CMH) Lahore Medical College, Lahore, PAK; 2 Internal Medicine, Combined Military Hospital (CMH) Lahore Medical College and Institute of Dentistry, Lahore, PAK

**Keywords:** von recklinghausen’s disease, inferior vena cava tumor thrombus, deep venous thrombosis (dvt), retroperitoneal neurofibroma, solitary neurofibroma

## Abstract

A neurofibroma is a benign, non-encapsulated neoplasm of the peripheral nerve sheath. These tumors are a notorious manifestation of the autosomal dominant condition known as neurofibromatosis type 1, where they present as multiple, cutaneous masses with high malignant potential. On the contrary, benign solitary retroperitoneal neurofibromas (SRN) occur without any associated conditions and have rarely been documented. Our case is of a 40-year-old male who presented with a three-month history of painful calf swelling, refractory to over-the-counter painkillers which was later diagnosed as deep vein thrombosis (DVT). A computed tomography (CT) angiogram was done which revealed a mass in the retroperitoneum impinging on the inferior vena cava (IVC). Approximately one month later, the whole mass was surgically excised and histopathology confirmed the diagnosis of a neurofibroma. This case presentation proved to be novel as it highlights the evaluation and management of a rare SRN which resulted in extensive DVT.

## Introduction

Nerve sheath tumors (NST) are soft tissue neoplasms that consist of benign and malignant schwannomas and neurofibromas and account for 4% of retroperitoneal tumors [1]. A neurofibroma is a slowly growing, non-encapsulated neoplasm of the peripheral nerve sheath [2]. They usually lack an identifiable parent nerve [1]. These tumors are equally present in both genders and affect adults between the 3rd and 6th decades of life [3]. Most cases of neurofibromas are associated with neurofibromatosis-type 1 (NF1), an autosomal dominant neurocutaneous syndrome encompassing a plethora of diagnostic manifestations, one of which are the presence of multiple cutaneous plexiform neurofibromas which have an increased risk of becoming malignant peripheral nerve sheath tumors (MPNST); MPNSTs are present in 2-5% of these patients [4]. Only rarely have these tumors been documented as solitary benign entities without any associated conditions [2]. Additionally, most neurofibromas are found on the skin, peripheral nerves, and paraspinal nerve roots [5]. Primary retroperitoneal tumors represent a rare, diverse group of neoplasms that arise within the retroperitoneal space, external to the major organs [6]. Considering these factors, a solitary retroperitoneal neurofibroma (SRN) is an exceptionally rare diagnosis as proven by the paucity of case reports published [3]. Clinically, many neurofibromas are asymptomatic but they may also present with vague, poorly localized pain or gastrointestinal discomfort [3]. Often, they come to attention only by the compressive symptoms they exert on surrounding structures which may include obstruction of the bowel, ureter, and biliary system resulting in colicky pain [1]. Although these tumors have a very low potential for malignant transformation, accurate diagnosis is essential to allow for adequate and prompt treatment as their presence in the abdominal cavity can result in many complications including obstructive jaundice, gastrointestinal bleeding, or colonic intussusception [5].

## Case presentation

A 40-year-old male presented to the emergency department with a three-month history of painful calf swelling in the left leg. The pain was persistent throughout the day during walking, climbing stairs, and at rest. Initially, he managed the pain with over-the-counter painkillers, but more recently, it became more excruciating and refractory to the pain killers. There were no other associated features including warmth and redness, shortness of breath, or chest pain. Physical examination was positive for enlarged, non-tender, matted inguinal, and popliteal lymph nodes on the left side approximating 1.5 cm in size; the rest of the examination was unremarkable.

A doppler ultrasound of the left leg revealed extensive thrombosis in the deep veins including external iliac, superficial and deep femoral, popliteal, and tibial veins causing almost complete obliteration. Ultrasound abdomen was also performed which showed a hypoechoic mass in the retroperitoneal space, just below the level of the aortic bifurcation. A CT angiogram of the abdomen and pelvis confirmed the presence of a well-defined mass in the retroperitoneum causing mass effect on inferior vena cava (IVC) and left common iliac vein with complete thrombosis of the left common iliac, left external iliac left common femoral, and left superficial femoral veins. The corresponding veins in the right leg were patent. The patient was referred to an interventional cardiologist for assessment of the venous blood clots for which he was prescribed enoxaparin (low-molecular-weight heparin (LMWH)).

**Figure 1:**
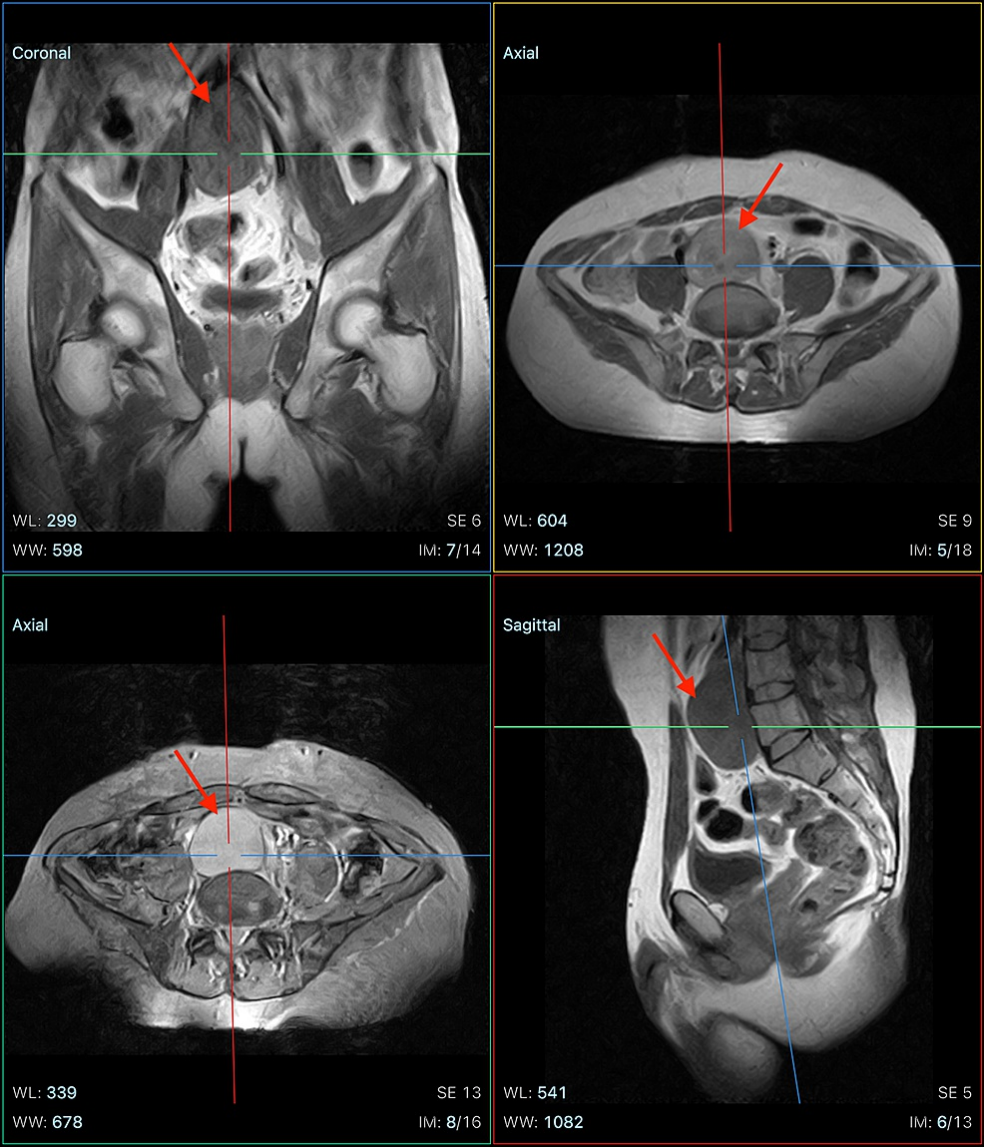
MRI pelvis showing a retroperitoneal mass in the prevertebral space, along L4 and L5 vertebrae, with dimensions of 7.5 cm × 6 cm × 5.5 cm (TV × AP × CC) in coronal (top left), axial (top right, bottom left), and sagittal (bottom right) views. MRI: magnetic resonance imaging; TV: transverse; AP: anteroposterior; CC: coronal

To further assess the mass, contrast-enhanced CT (CECT) of the abdomen and pelvis was ordered, which revealed a well-defined, low-density homogenous midline abdominal mass measuring 7.5 cm × 6 cm × 5.5 cm (TV × AP × CC), located in the infra-aortic retroperitoneal plane in the prevertebral space, anterior to the L4 and L5 vertebrae, and posterior to the iliac vessels. Further assessment with magnetic resonance imaging (MRI) with contrast likewise highlighted a well-defined heterogeneously enhancing lesion in the prevertebral space along the L4 and L5 vertebrae (Figure 1).

**Figure 2:**
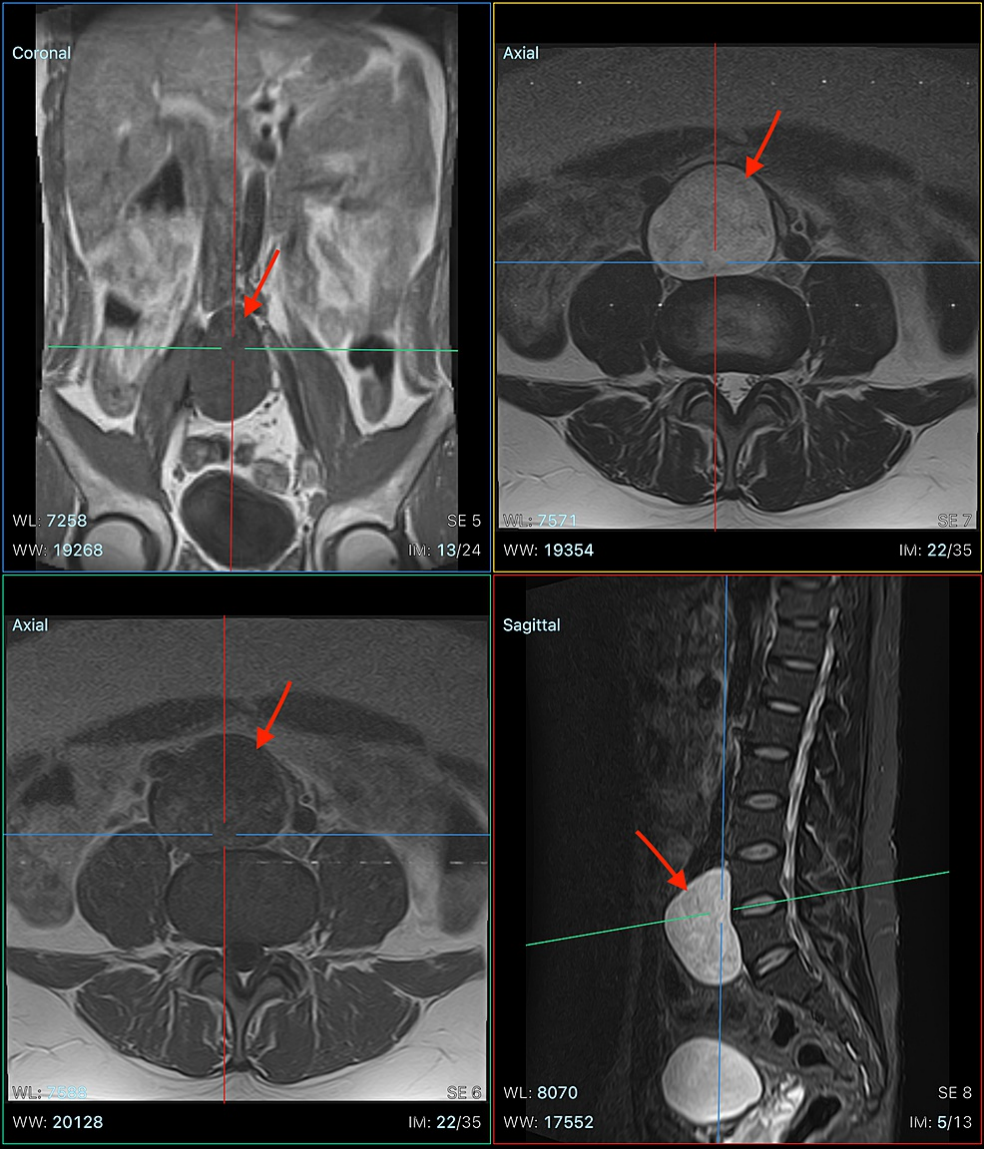
MRI lumbosacral spine showing a retroperitoneal mass isolate from spinal cord structure in coronal (top left), axial (top right, bottom left), and sagittal (bottom right) views. MRI: magnetic resonance imaging

The patient was then assessed for whether a laparoscopic biopsy could be performed in order to identify the mass. Unfortunately, due to the sensitive location of the mass-as revealed by the MRI-a laparoscopic biopsy was not conducted. Instead, further assessments were conducted, which included an MRI spine which was to assess whether the mass was an extension of the dura mater (Figure 2). Imaging revealed that the mass was isolate and separate from the spinal cord structure.

At this time, the only management advised was surgical removal of the mass. Over the next month, the patient eagerly searched for a specialized surgeon to perform the procedure while also continuing enoxaparin for his deep vein thrombosis (DVT). After consulting numerous physicians, the patient’s case was finally taken by a general surgeon at a government hospital in Lahore, Pakistan, and surgical excision of the mass was planned.

**Figure 3:**
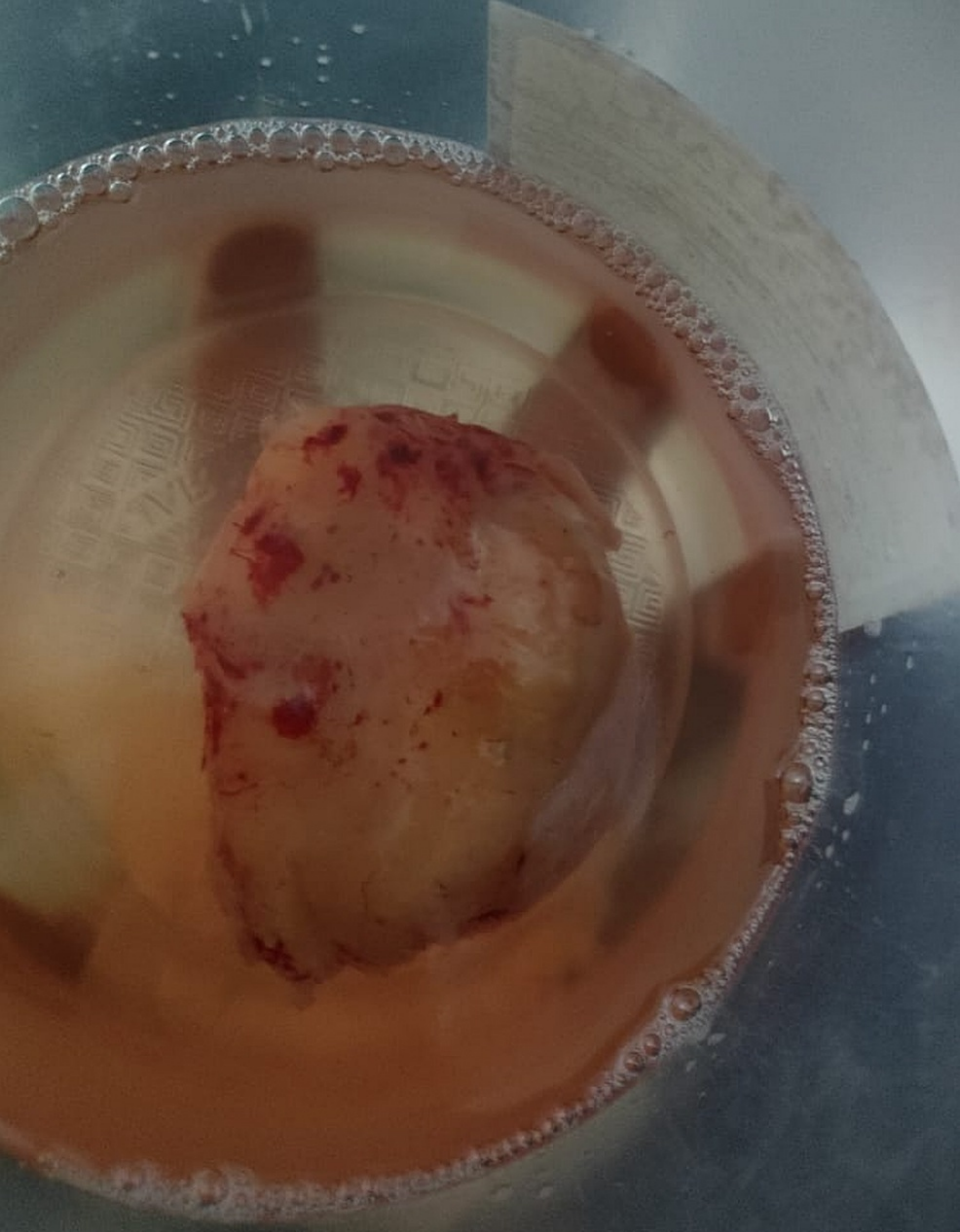
Gross image of well-circumscribed, skin-tan colored mass, with firm consistency, measuring 7.5 cm × 6 cm × 5.5 cm (TV × AP × CC); image taken after surgical excision. TV: transverse; AP: anteroposterior; CC: coronal

Enoxaparin was discontinued 24 hours prior to the procedure. An IVC filter was placed and a cardiothoracic surgeon was on standby in case of intraoperative complications such as pulmonary embolism. The surgery was performed within an hour and 30 minutes and the complete mass was successfully excised (Figure 3).

**Figure 4:**
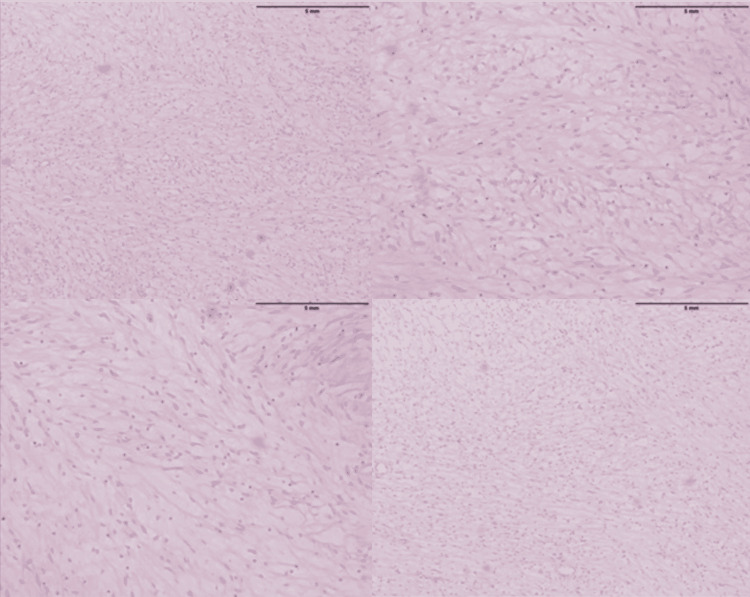
Histopathology across multiple cross-sections revealing a partially encapsulated lesion composed of a monotonous population of spindle-shaped cells with wavy nuclei.

At this time, a differential diagnosis was made which included the following: neurofibroma, schwannoma, lymphoma, liposarcoma, and rhabdomyosarcoma. In the postoperative period, the patient maintained bed rest without any anticoagulants. Enoxaparin was resumed three days after the operation. Histopathology of the mass across multiple cross-sections was performed revealing a partially encapsulated lesion composed of a monotonous population of spindle-shaped cells with wavy nuclei (Figure 4).

**Figure 5:**
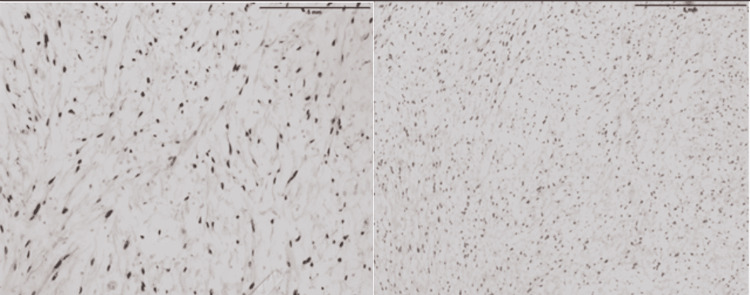
Histopathology with SOX10 immunostain showing nuclear staining in tumor cells in two separate cross-sections.

The background encompassed thin-walled blood vessels. Anaplastic features were not apparent. The tumor was SOX10: positive, DOG1: negative, CD117: negative, and desmin: negative (Figure 5).

A diagnosis of neurofibroma was confirmed in the report. Because of the strong association of neurofibromas with NF1, relevant medical history and physical examination for NF1 and NF2 were completed, both negative. Additionally, family history for NF1 or any other genetic syndrome was also negative.

## Discussion

Neurofibromas are the most prevalent benign peripheral NSTs [7]. They account for 5% of all benign soft tissue tumors [8]. Our case presents the findings of a rare variant that was not only sporadic but also retroperitoneal. To further add to the rarity of this case, it is perhaps the first of its kind in which the tumor was an incidental finding after investigating the patient’s initial presenting complaint of calf pain, which was diagnosed as DVT. Our case can be compared with other similar cases. Almarie et al. highlighted the management of a 43-year-old woman diagnosed with a localized retroperitoneal pararenal neurofibroma [5]. Njoumi et al. published a case of a 29-year-old male with a solitary *preperitoneal* neurofibroma [9]. Other case reports have reported even more distinct locations of neurofibromas including the hard palate [10], the inguinal region [8], and the retroperitoneal pelvic area [11]. Considering the above, it is evident that solitary neurofibromas have the propensity to develop anywhere in the body.

The classic manifestations of solitary neurofibromas are not explicit and vary according to their respective locations [9]. Most are discovered as a result of their impingement on contiguous structures which may result in palpable masses, abdominal pain which may be colicky, and transit disorders owing to extraluminal pressure [9]. Our patient initially presented with symptoms of DVT, for which he didn’t have any underlying risk factors. Further investigations revealed a retroperitoneal mass closely applied to the bifurcation of the IVC, causing extensive thrombosis of the left common iliac vein and extending into the lower limb veins. Only a few other cases with similar presentations were found. Theodosopoulos et al. reported the findings of a 53-year-old male who presented with DVT of the left leg, which evidently turned out to be caused by a retroperitoneal schwannoma [12]. Similarly, Wee-Stekly et al. presented a case of a 28-year-old female who presented with traction pain in both legs while walking, which was caused by a retroperitoneal myxoid neurofibroma in the left presacral area [11]. On the contrary, Taketomi et al. published the findings of a 24-year-old woman who presented with three months history of asymptomatic mass on the hard palate which was later diagnosed as a solitary neurofibroma [10]. Evidently, neurofibromas present with a wide range of clinical manifestations and are usually incidental findings that become symptomatic only when they enlarge sufficiently to impinge on surrounding structures [11]. For any large solitary mass with slow growth and no signs of malignancy, a neurofibroma should always be included in the differential [8]. This, along with schwannoma, liposarcoma, and lymphoma made up our differential diagnosis.

Regardless of the severity of presenting symptoms, efforts should always be made for prompt diagnosis of any suspected retroperitoneal mass in order to prevent detrimental complications [5]. Late diagnosis of intra-pelvic tumors may also lead to surgical resection difficulties and an overall poor prognosis [8]. For example, in the previously mentioned case of retroperitoneal schwannoma resulting in DVT, an exploratory laparotomy was done, only to find that the tumor was heavily adherent to the sacrum and also displaced the bladder and recto-sigmoid colon, which prevented excision at that time and re-exploration at a later date was necessitated [12].

The diagnosis of neurofibromas depends on various imaging modalities with MRI and CT scans being the most widely utilized [8]. Preoperative imaging, however, is usually insufficient to diagnose with certainty and a biopsy is typically warranted for confirmation with histopathology [9]. However, in some cases, a preoperative biopsy is not plausible due to the sensitive location of the tumor as seen on imaging. This was the case for our patient whose tumor was of significant size and located in the vulnerable prevertebral space in close proximity to the iliac vessels. In the patient with retroperitoneal schwannoma, there were similar concerns and a core needle biopsy was not attempted because the patient was taking anticoagulants for his DVT [12]. In our case, the inability to carry out preoperative biopsy did not only provide difficulty in diagnosis, but it also incurred significant distress for the patient, who spent over a month trying to find a specialized surgeon willing to operate on an undiagnosed mass. In these cases, other investigations may be conducted in an attempt to further enhance the identification of the mass. For our patient, he underwent an MRI scan of the spine which revealed that the mass was isolate and separate from the spinal cord structure. Lastly, because neurofibromas have a strong association with NF1, clinical manifestations and necessary investigations should always be carried out to exclude the possibility of malignancy [8].

For neurogenic tumors, including schwannomas and neurofibromas, early surgical removal is warranted and patients generally have an excellent prognosis post-resection [8]. Surgical removal entails en-bloc resection without the hindrance of the capsule [5]. Many neurofibromas have been reported as difficult to surgically manage owing to their dimensions, widespread infiltration, and generous vascularity [9]. Occasionally, some surgeons may choose to only operate under the circumstance that the tumor is causing distress, neurological deficiencies, or is carrying high malignant potential. However, as mentioned earlier, the prolonged presence of any unidentified mass in the body has the capacity to impose detrimental effects and, therefore, surgical removal should always be considered as first-line. Additionally, it is important to take necessary precautions during surgery depending on individual patient risks. In our patient, as well as in the patient with retroperitoneal schwannoma, both presented with DVT and required an IVC filter during surgery [12]. Postoperatively, our patient recovered well with no complications. There were no signs of recurrence on follow-up.

The gross and histopathological features of neurofibromas vary considerably, but most are described as well-circumscribed with an incomplete capsule and a grey-tan color [7]. These tumors consist of fibroblasts, Schwann cells, and neural elements [13]. Microscopically, they are usually arranged as loose hap-hazard spindle-shaped cells with ill-defined cell borders posing on a background of myxoid to pale pink collagenous matrix [7]. Our patient’s biopsy was done after excision of the tumor and likewise, it revealed a partially encapsulated lesion composed of a monotonous population of spindle-shaped cells with wavy nuclei on a background of thin-walled blood vessels and without any anaplastic features.

## Conclusions

SRNs are rarely encountered in the clinical setting resulting in a lack of related literature. For this reason, they are often overlooked or difficult to identify. In general, any large solitary retroperitoneal mass that grows slowly and produces any sort of compression symptoms should be a suspected neurofibroma. This rule can help direct our focus in the clinical setting if a patient presents with arbitrary symptoms such as our patient who presented with DVT and enlarged inguinal lymph nodes. The appropriate diagnosis and treatment of neurofibromas depend on the collaborative efforts of a multidisciplinary healthcare team. Effective communication between concerned specialties can avoid unnecessary delays in diagnosis. Lastly, more elaborate studies are warranted in order to identify standards of safe management, especially for cases like ours where a mass could not be diagnosed until its surgical intervention.
